# A Circular Microstrip Antenna Sensor for Direction Sensitive Strain Evaluation †

**DOI:** 10.3390/s18010310

**Published:** 2018-01-20

**Authors:** Przemyslaw Lopato, Michal Herbko

**Affiliations:** Department of Electrical and Computer Engineering, West Pomeranian University of Technology, ul. Sikorskigo 37, Szczecin 70-313, Poland

**Keywords:** microstrip sensor, microstrip antenna, microwave technique, structural health monitoring, deformation measurement, strain measurement, finite element method

## Abstract

In this paper, a circular microstrip antenna for stress evaluation is studied. This kind of microstrip sensor can be utilized in structural health monitoring systems. Reflection coefficient *S*_11_ is measured to determine deformation/strain value. The proposed sensor is adhesively connected to the studied sample. Applied strain causes a change in patch geometry and influences current distribution both in patch and ground plane. Changing the current flow in patch influences the value of resonant frequency. In this paper, two different resonant frequencies were analysed because in each case, different current distributions in patch were obtained. The sensor was designed for operating frequency of 2.5 GHz (at fundamental mode), which results in a diameter less than 55 mm. Obtained sensitivity was up to 1 MHz/100 MPa, resolution depends on utilized vector network analyser. Moreover, the directional characteristics for both resonant frequencies were defined, studied using numerical model and verified by measurements. Thus far, microstrip antennas have been used in deformation measurement only if the direction of external force was well known. Obtained directional characteristics of the sensor allow the determination of direction and value of stress by one sensor. This method of measurement can be an alternative to the rosette strain gauge.

## 1. Introduction

Nowadays, there is very rapid development of wireless technologies, which are inherent in microwave circuits [1,2,3]. In addition, the high-tech industry requires small mass communication equipment that is easy to install, has good aerodynamic profile, high-performance and is cheap to produce. For these reasons, microstrip antennas and other microstrip elements are developed. The idea of a microstrip antenna can be traced to 1953 [1]. Due to the directionality of the radiation pattern caused by the ground plane and the ease of integration of such antennas with electronic circuits (with antenna and transceiver power lines), this type of antenna has been used in mobile phones. In this application, the ground plane limits head radiation.

In the last decade, microstrip structures have also been used outside of communication applications. During this time, many ideas of microstrip sensors have come up, to measure various physical parameters. One of them is a sensor for the thickness and dielectric constant of solid and liquid materials evaluation [4]. Another interesting application for non-contact liquid sensing using capacitive coupled planar ring resonators was presented in [5]. In literature, a microstrip microfluidic sensor has been reported [6]. Microstrip transducers have also been used in gas sensing [7,8], pressure monitoring [9] and temperature evaluation [10,11,12,13]. Microwave double ring resonator was utilized to investigate the permittivity and conductivity variation on the monolayer coated nanotube membrane [14]. Another applications of microstrip sensors include: glucose monitoring [15] and assessment of meat aging [16]. Moreover, microwave transducer was used for measurement of angular velocity and angular displacement [17]. It can be alternative for optical encoders, Hall effect sensors, RVDT and rotation sensor based on magnetic microrods [18].

Microstrip sensors were also used to measure mechanical properties such as shear [19], crack [20,21] and strain [22,23,24,25,26,27,28,29,30,31,32,33,34,35]. The resonance frequency shift is used by most microstrip sensors to study physical parameters. The resonance frequencies measured by Vector Network Analysers (VNAs).

Until recently, VNA prices were very high. However, there has been an emergence of low-cost Vector Network Analysers (for example pocketVNA or miniVNA Tiny) for the frequencies below 3 GHz. In addition, the current advances in microwave integrated circuits technology and their mass production, allows us to state that: soon, the cost of a Structural Health Monitoring (SHM) system using microstrip sensors will be competitive compared to other technologies. One of the cost-effective solutions for the development of microwave systems (for measurements of physical quantities), are Frequency Modulated Continuous Wave (FMCW) based interrogation circuits. In this case resonant frequency can be measured using portable and battery-operated devices [36]. In addition, the antenna sensors have one unique property, which is the possibility of wireless interrogation without using a battery operation. This approach can be useful in harshly conditions and testing of moving parts. For this reason, various methods of wireless measurements were developed:

- RFID system based on passive sensors and an ultra-wideband (UWB) reader [10],
- passive structure (e.g., dielectric resonator) is both a sensor and an interrogated element [11,12],
- wireless sensor node consists of two antennas (microstrip antenna utilized as the transmission/receiving (Tx/Rx) device and a separate microstrip patch structure serving as the temperature-sensing element) connected by a transmission line [13].

These days, sensors for monitoring the state of structures are very important. This is due to the development of the Structural Health Monitoring technique. This method is used to monitor the state of construction of space vehicles, aircraft and civil structures. Increasingly, SHM systems are replacing or supporting traditional inspection methods. SHM systems can be more effective than periodical inspection because it enables the overseeing of a structure in real time. Thus, the SHM technique provides a high level of safety and decreases the maintenance cost [37,38]. In the case of monitoring the state of aircraft fuselage, an additional advantage is the reduction of service time [39]. One of the parts of the SHM system is the sensor network. Sensors for monitoring various parameters of the structure are used, but the key elements of the system are strain sensors. Approximately 50% of all sensors in case of bridge monitoring systems are strain sensors [37]. Until now, stress measurements were performed using strain gauges, piezoelectric sensors, magnetic sensors and fiber optic sensors [40,41,42,43,44,45,46]. In recent years, there has been a lot of research on the use of microstrip antennas for stress evaluation [22,23,24,25,26,27,28,29,30,31,32,33,34,35]. Deformation and strain assessment by microstrip antenna relies on *S*_11_ coefficient investigation. This type of sensor is attached to the evaluated element, so its deformation causes a change in the sensor’s geometry. This behaviour introduces a shift of resonant frequency Δ*f_r_*:

(1) $$\Delta f_{r}=f_{r0MPa}-f_{rload}$$

where: *f_r_*_0*MPa*_—resonant frequency for 0 MPa stress (no load), *f_rload_*—resonant frequency for setup with load.

Until now, the most frequently studied microstrip sensor was the one based on rectangular patch [23,24,25,26,30,31,33,34]. In addition, circular microstrip antennas [29,35] and other shapes of patch [22,27,28] for deformation evaluation were investigated. A microstrip sensor has a linear dependence of the resonant frequency shift with deformation. However, the change of resonant frequency for different angles of external force excitation is not the same. Until now, strain directional characteristics have not been determined for microstrip antennas. For a sensor with a rectangular patch, two resonant frequencies in two orthogonal directions were tested [23,24,25,26,30,34]. In the case of the circular microstrip sensor [29] and with other shapes of patch [22,27,28], one resonant frequency was tested for three different stress angles. 

In this work, the concept of direction sensitive deformation sensing introduced in our previous research [35] is studied more detailed. New circular microstrip antenna sensor was designed and fabricated. Stress/strain directional characteristics for two resonance frequencies of the obtained resonator were determined using finite element method model. These characteristics allow for simultaneous measurement of both the direction and values of stress using a single sensor. So far, the direction and value of stress have been tested by strain gauge rosette or fibre optic strain gauge rosette [44], because the directional characteristics of microstrip stress sensors have not been determined. Finally, an experiment was conducted for verification of obtained stress directional characteristics.

## 2. Sensor Design

**Figure 1:**
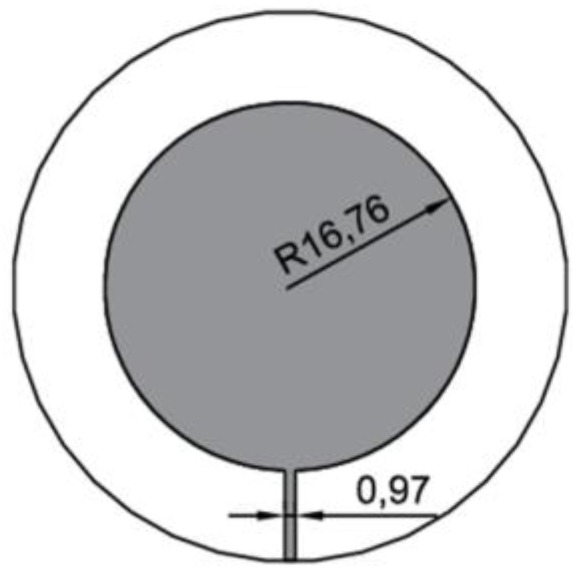
View and dimensions (in mm) of designed sensor.

Various configurations can be utilized to feed microstrip structure: aperture or proximity coupling, microstrip or coaxial transmission line. Because of fabrication complexity issues and ease of adhesive connection to deformed structure, sensor designed in this work is fed by microstrip line. First, circular microstrip structure was designed using cavity model, for operating frequency of *f_r1_* = 2.5 GHz at fundamental mode and impedance of 50 Ω. The obtained dimensions are presented in Figure 1. The double-side polymer laminate (relative permittivity of 4.4) of the thickness 0.5 mm was used to design circular microstrip antenna using following equation [1]:

(2) $$R=\frac{F}{{\left\{1+\frac{2h}{\pi \varepsilon_{r}F}\left[\ln\left(\frac{\pi F}{2h}\right)+1.7726\right]\right\}}^{\frac{1}{2}}}$$

where:

(3) $$F=\frac{8.791\times 10^{9}}{f_{r}\sqrt{\varepsilon_{r}}}$$

*R*—radius of patch, *ε_r_*—relative permittivity of substrate (*ε_r_* = 4.4), *f_r_*—resonant frequency, *h*—height of substrate.

## 3. Numerical Analysis

Comsol Multiphysics environment was utilized to develop FEM (Finite Element Method) numerical model and for assessment of designed sensor. In order to obtain deformation of steel sample and sensor geometry, Solid mechanics module was applied. Thereafter, the RF module allowed calculation of *S*_11_ coefficient for deformed sensor. 

**Figure 2:**
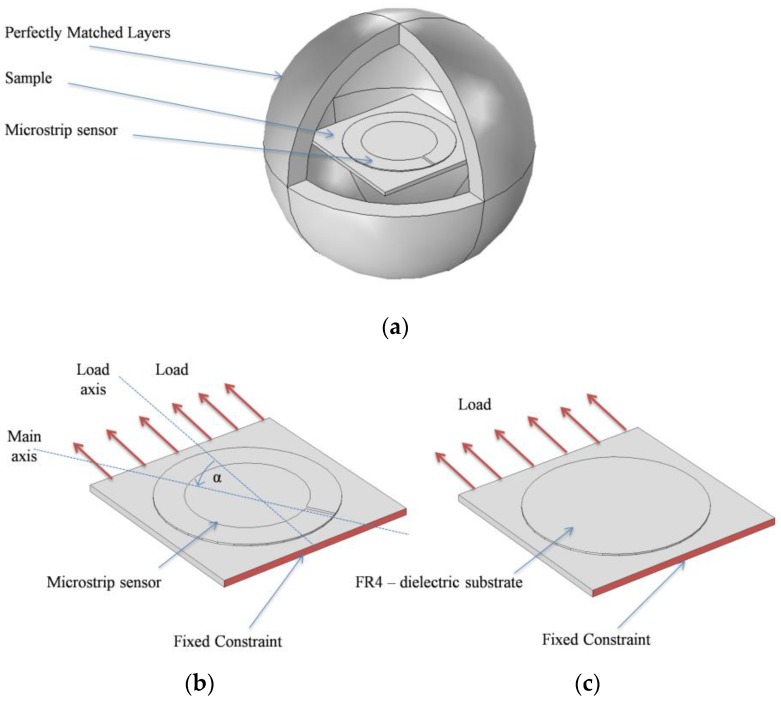
Finite Element Method (FEM) numerical model; (**a**) entire numerical model for directional characteristics of the microstrip sensor determination; (**b**) top view of the studied sample; (**c**) bottom view of the tested sample.

**Figure 3:**
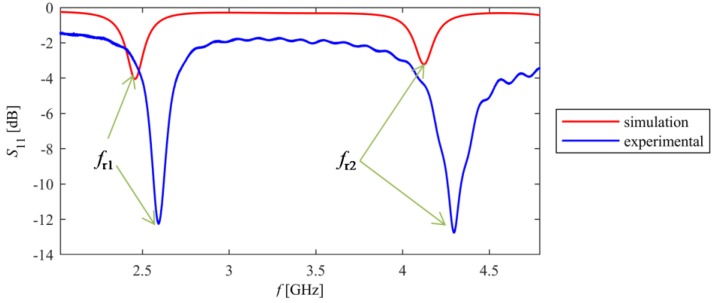
Reflection coefficient for designed sensor.

**Figure 4:**
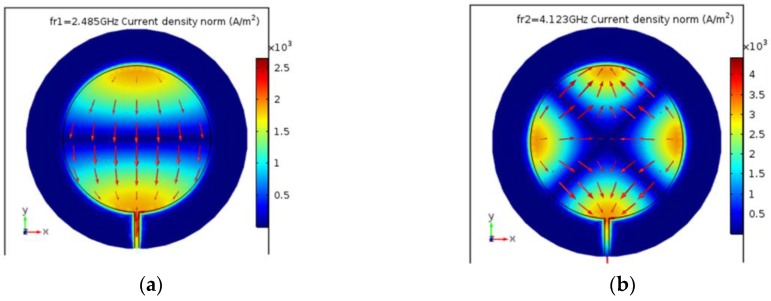
Current distribution and density in patch; (**a**) first resonant frequency *f_r1_*; (**b**) second resonant frequency *f_r2_*.

The study consists of two parts. In the first part, directional characteristics of deformation were calculated. For this purpose, the microstrip sensor was attached to a sample from one side, whereas the same laminate was attached to the opposite side of the sample. This configuration provides only planar deformation to sensor (without this, there is a bending behaviour of the sample). The numerical model is shown in Figure 2. The computational domain contains Perfectly Matched Layers (PML)—an exterior domain of the model (having complex-valued and anisotropic permeability and permittivity) that absorbs outgoing waves emitted by microstrip antenna sensor. Utilized design of PML domain is based on information provided by the manufacturer of the Comsol Multiphysics environment. The directional characteristics were determined with step of 10 degrees for eight stress levels (from 0 to 350 MPa with a 50 MPa step). The sample was made of S355J2+N steel, which parameters are presented in Table 1. The reflection coefficient of designed antenna sensor is presented in Figure 3. One can observe two resonant frequencies *f_r1_* and *f_r2_*. Differences between the results obtained in the simulations and measurements are caused by variation in the electromagnetic properties of laminate (the exact value of the dielectric permittivity was not measured for the utilized material). This ambiguity causes a difference in measured and simulated resonant frequencies, however, it does not significantly affect the transducer's performance, because the deformation (and stress) are determined on the basis of the frequency shift Δ*f_r_*. In Figure 4, current distribution and density in patch for both resonant frequencies are shown.

**Figure 5:**
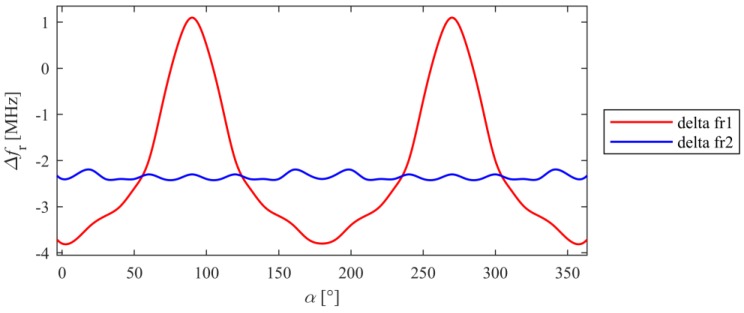
Directional characteristics of resonant frequency shift caused by stress of 350 MPa.

**Figure 6:**
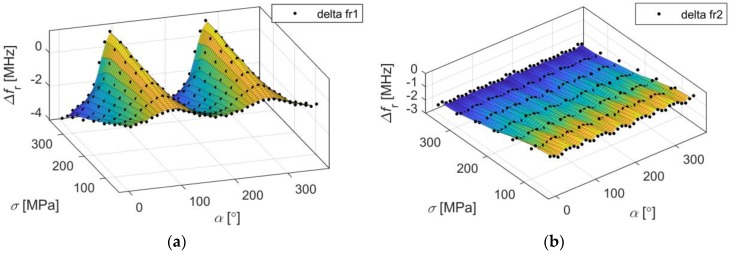
Directional characteristics of resonant frequency shift for different stress level (**a**) first resonant frequency; (**b**) second resonant frequency.

For selected resonant frequencies and changes of load directions, strain directional characteristics were calculated. Characteristics determined for *f_r1_* and *f_r2_* are shown in Figure 5 and Figure 6. Based on presented results it may be stated that the shift of the first resonant frequency *f_r1_* is much more variable than the shift of the second resonant frequency *f_r2_*. It is possible to receive higher Δ*f_r_* values for orthogonal directions, but for some other ones the sensor is completely insensitive for strain (Δ*f_r1_* = 0 MHz). In case of the second resonance, similar sensitivity for stress level for all direction of the loading force was obtained. This resonant frequency can be used for deformation evaluation even if the direction of potential load is unknown. The reason is that current distribution in the patch for *f_r2_* is more multidirectional as shown in Figure 4.

**Figure 7:**
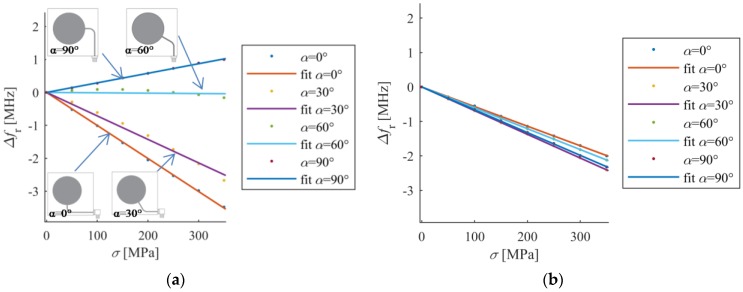
Shift of resonant frequencies for different stain angle (simulation results), (**a**) first resonant frequency; (**b**) second resonant frequency.

In the second part of the study shift of resonant frequency for different stress levels was examined. Four sensors were designed for various angles of microstrip line feed (0°, 30°, 60° and 90°) which mimics various angles of deformation treatment. In case of four considered directions and different stress level, Δ*f_r_* was calculated. The results of this numerical analysis were presented in Figure 7. All dependencies are linear, because the yield point was not exceeded. In the case of a second resonant frequency all lines have approximately the same value of gradient in contrast to first resonant frequency, where depending on direction of deformation Δ*f_r1_* can be increased (α = 90°), decreased (α = 0°, α = 30°) or unchanged (α = 60°). Thus, the second resonant frequency is less sensitive on deformation direction as shown in Figure 7, and can be utilized to detect any direction of deformation. Moreover, the first resonant frequency for *α* = 60° is not sensitive on deformation (Figure 7), therefore single *f_r1_* resonance should not be utilized if direction of mechanical excitation is not known or may change.

## 4. Experimental Analysis

**Figure 8:**
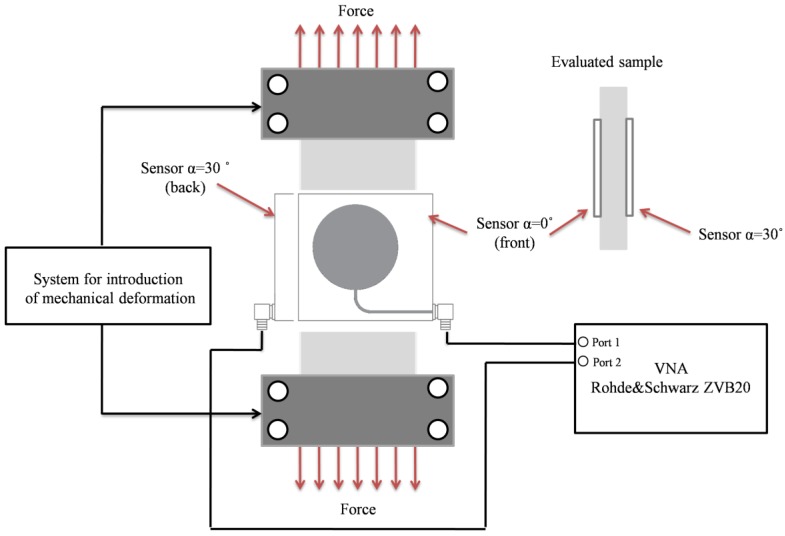
Simplified scheme of proposed measurement system.

**Figure 9:**
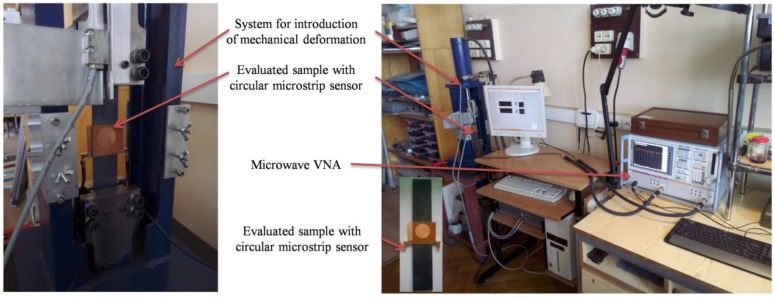
Photo of measurement system for evaluation of deformation produced under static loading conditions.

The results of numerical analysis shown in Figure 7 (shift of resonant frequency for different stress levels) were verified by measurements. Based on the investigated numerical models, four transducers were manufactured using photolithographic process. The sensors were adhered with cyanoacrylate adhesive to the sample as illustrated in Figure 8 and Figure 9. This adhesive connection allows transmission of sample deformation to microstrip sensor. The sample was deformed by introduction of mechanical stress. The S355J2+N steel sample dimensions were as follows: length 2500 mm, width 45 mm and thickness 2 mm. Measurements were carried out using the Rohde & Schwarz ZVB20 vector network analyser in 2–5 GHz frequency range with step of 0.3 MHz. The simplified scheme and photo of utilized measurement system are shown in Figure 8 and Figure 9 respectively. During the experiment, two sensors were utilized at one time (adhered on opposite sides of the sample and connected to separate ports of VNA).

**Figure 10:**
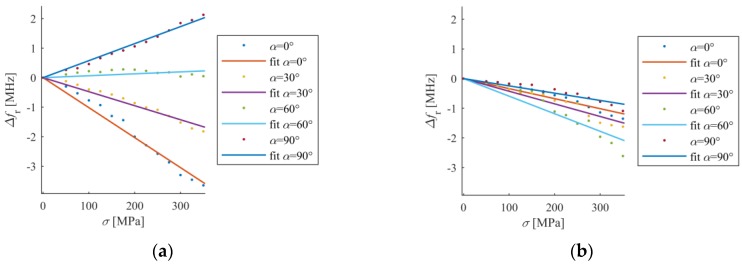
Shift of resonant frequencies for different stain angle (experimental results), (**a**) first resonant frequency; (**b**) second resonant frequency.

The *S*_11_ frequency responses obtained in measurement and simulation vary due to lack of knowledge of the exact properties of utilized FR4 laminate (Figure 3). Figure 10a shows the relationship between stress and first resonant frequency shift for various deformation angles, whereas Figure 10b presents this relationship for second resonant frequency. All characteristics are nearly linear, as predicted by simulations.

First resonant frequency is more sensitive to direction of load than the second one (Figure 10). Furthermore, first resonant frequency is not sensitive on strain for α = 60° (Figure 10a). A good convergence between the results in the simulations and measurements was obtained. Small differences in gradient of received characteristics between numerical and experimental analysis can be caused by variation in the electromagnetic properties of laminate and mechanical properties of transducer-sample adhesive connection (which was assumed as ideal one during numerical simulations).

## 5. Conclusions

In this paper, strain directional characteristics for two resonant frequencies of circular microstrip sensor was considered, which was not studied for any microstrip sensor so far. It should be pointed that monitoring of two resonant frequencies is essential and allows strain direction assessment using circular microstrip sensor. This is especially important in cases when sensor is totally insensitive for external stress (like in case of α = 60°). Furthermore, this type of sensor can be a good alternative for strain gauge, because one microstrip sensor (by analysing two resonances) can provide the information about the value and direction of deformation, while these parameters can be determined by three separate strain gauges.

## Figures and Tables

**Table 1:** Mechanical properties of S355J2+N steel according to EN 10025.

Steel S355J2+N Parameters	
Young’s modulus E	200 GPa
Yield point	355 MPa
Limit state	510 MPa
